# Reciprocal activation between IL-6/STAT3 and NOX4/Akt signalings promotes proliferation and survival of non-small cell lung cancer cells

**DOI:** 10.18632/oncotarget.2671

**Published:** 2015-01-08

**Authors:** Juan Li, Tian Lan, Cuixiang Zhang, Cheng Zeng, Jincai Hou, Zhicheng Yang, Min Zhang, Jianxun Liu, Bing Liu

**Affiliations:** ^1^ Clinical Pharmacy Department, Guangdong Pharmaceutical University, Guangzhou 510006, China; ^2^ Vascular Biology Research Institute, Guangdong Pharmaceutical University, Guangzhou 510006, China; ^3^ Institute of Basic Medical Sciences of Xiyuan Hospital, China Academy of Chinese Medical Sciences, Beijing 110300, China; ^4^ Beijing Key Laboratory of Pharmacology of Chinese Materia Medica, Beijing 110300, China; ^5^ Department of Pharmacology, School of Pharmacy, Guangdong Pharmaceutical University, Guangzhou 510006, China; ^6^ Department of Health Statistics, School of Public Health, Guangdong Pharmaceutical University, Guangzhou 510006, China

**Keywords:** NOX4, IL-6, non-small cell lung cancer, oxidative stress

## Abstract

Inflammatory cytokines and oxidative stress are two critical mediators in inflammation-associated cancer. Interleukin-6 (IL-6) is one of the most critical tumor-promoting cytokines in non-small cell lung cancer (NSCLC). In our recent study, we confirmed that NADPH oxidase 4 (NOX4), an important source of reactive oxygen species (ROS) production in NSCLC cells, promotes malignant progression of NSCLC. However, whether the crosstalk of NOX4 and IL-6 signalings exists in NSCLC remains undentified. In this study, we show that NOX4 expression is positively correlated with IL-6 expression in NSCLC tissues. Exogenous IL-6 treatment significantly enhances NOX4/ROS/Akt signaling in NSCLC cells. NOX4 also enhances IL-6 production and activates IL-6/STAT3 signaling in NSCLC cells. Specifically, NOX4 is confirmed to functionally interplay with IL-6 to promote NSCLC cell proliferation and survival. The *in vivo* results were similar to those obtained *in vitro*. These data indicate a novel NOX4-dependent link among IL-6 in the NSCLC microenvironment, oxidative stress in NSCLC cells and autocrined IL-6 in NSCLC cells. NOX4/Akt and IL-6/STAT3 signalings can reciprocally and positively regulate each other, leading to enhanced NSCLC cell proliferation and survival. Therefore, NOX4 may serve as a promising target against NSCLC alone with IL-6 signaling.

## INTRODUCTION

Chronic inflammation is induced by a variety of pathogenic and environmental factors and is associated with an increased risk of several human cancers [1]. The lung is vulnerable to numerous pathogens and polluted gases, and persistent exposure to these factors results in release of various inflammatory cytokines from inflammatory cells, leading to chronic inflammation and risks of lung cancer [2, 3].

Chronic inflammation has also been linked to cancer survival and proliferation [4]. Inflammatory cells in the tumor microenvironment may release cytokines to directly stimulate oncogenic signaling in cancer cells, including NF-κB, STAT3 and HIF1α signalings, resultantly promoting cancer survival and proliferation [5]. Alternatively, cytokines can also stimulate reactive oxidative species (ROS) accumulation in neighboring epithelial cells. For example, IFN-γ and LPS synergistically increase ROS production in human pancreatic cancer cell lines [6]. ROS have been reported to promote tumor cell survival and growth through activation of various signal pathways in cancer cells [7, 8]. NADPH oxidases (nicotinamide adenine dinucleotide phosphate oxidase, NOXs) are among the best-characterized cellular oxidases that generate ROS [9]. NOXs consist of seven members, represented by different catalytic subunits: NADPH oxidase 1 (NOX1), NOX2 (gp91^phox^), NOX3, NOX4, NOX5, Duox1, and Duox2 [10]. Many studies including ours have confirmed the close correlation of NOXs with cancer cell growth and malignant progression [11–13]. However, the molecular mechanisms underlying the associations among microenvironmental cytokines, ROS production in tumor cells and cancer promotion are not well defined.

One of the most critical tumor-promoting cytokines in non-small cell lung cancer (NSCLC) is interleukin-6 (IL-6). High levels of IL-6 in exhaled breath condensate (EBC) and in serum were confirmed to be related to tumor size in patients with NSCLC [14]. Circulating IL-6 level is also a prognostic marker for survival in advanced NSCLC patients or those treated with chemotherapy [15]. Targeting IL-6 signaling via blocking the IL-6R and IL-6 with siRNA or mAbs resulted in reduced H460 cell (a cell line of human NSCLC) proliferation [16]. On the other hand, human lung adenocarcinomas cells can produce their own IL-6 to promote cell proliferation [17]. A previous study indicates that NOX4 mediates production of various pro-inflammatory cytokines including IL-6 in renal cell carcinoma cells [18]. Our recent study shows that NOX4 is upregulated in NSCLC tissues and cells and promotes NSCLC cell proliferation and progression [19]. Nevertheless, whether the crosstalk of NOX4 and IL-6 signalings exists in NSCLC remains undentified.

In this study, we first found that NOX4 expression is positively correlated with IL-6 expression in NSCLC tissues. Human recombined IL-6 treatment could significantly enhance NOX4 expression and ROS production in NSCLC cells. NOX4 overexpression could also enhance IL-6 expression and stimulate its downstream signaling in these cells. Therefore, NOX4/Akt and IL-6/STAT3 signalings can reciprocally and positively regulate each other, leading to enhanced NSCLC cell proliferation and survival.

## RESULTS

### NOX4 expression is positively correlated with IL-6 expression in NSCLC tissues

**Figure 1:**
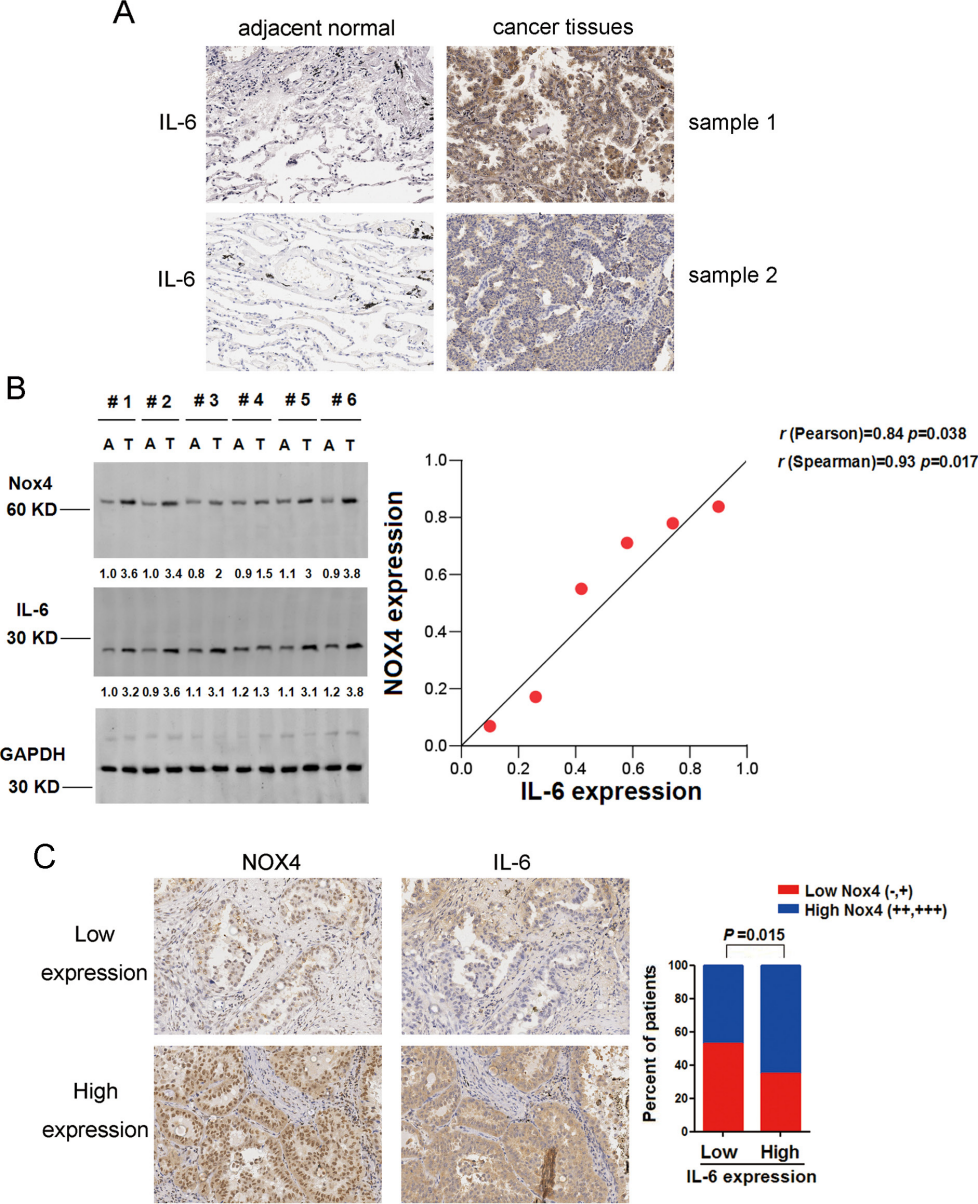
NOX4 is positively correlated with IL-6 levels of NSCLC **(A)** IHC staining indicating that IL-6 expression is upregulated in human NSCLC compared with adjacent normal lung tissues. **(B)** Western blotting analysis of NOX4 expression in 6 paired primary NSCLC tissues (T) and matched adjacent nontumor tissues (A). GAPDH was used as a loading control. **(C)** NOX4 expression associated with IL-6 expression in 152 primary human NSCLC specimens. Representative specimens with low and high levels of NOX4 are shown.

We first performed immunohistochemistry assay to determine the expression of IL-6 in NSCLC specimens. Immunohistochemical analysis showed that IL-6 was highly expressed in about 61% of NSCLC samples (93 of 152), whereas the adjacent normal tissues of NSCLC had much lower levels of IL-6 expression (Fig. 1A). The results of the IHC analysis are summarized in Table 1. We also collected 6 paired primary NSCLC tissues and matched adjacent nontumor tissues from the same patient and performed Western blotting in parallel to determine NOX4 expression. The results showed that NOX4 was elevated in all human NSCLC samples compared with the matched adjacent nontumor tissues (Fig. 1B). As a previous study indicates that NOX4 mediates hypoxia-induced IL-6 production in renal cell carcinoma cells [18], we next sought to the possible correlation of NOX4 and IL-6 expression levels in NSCLC. We found that IL-6 was also elevated in these NSCLC samples compared with the matched adjacent nontumor samples. In addition, statistical analysis showed a Spearman correlation coefficient of 0.93 (*p* = 0.017) and a Pearson correlation coefficient of 0.84 (*p* = 0.038) when the relative level of NOX4 expression was plotted against the relative level of IL-6 expression in these samples, suggesting a significant positive correlation between NOX4 and IL-6 expression in these samples (revised Fig. 1B). As shown in Fig. 1C, the clinical correlation studies in 152 specimens also showed that NOX4 levels were positively correlated with the expression of IL-6. The results of the IHC analysis are summarized in Table 2.

**Table 1:** Overexpression of IL-6 in human NSCLCs

		IL-6 expression No(%)		
	Numbers	(−,+)	(++,+++)	*p* value
Ajacent non-tumor tissues	115	68(59.1)	47(40.9)	0.0047
NSCLCs	152	59(38.8)	93(61.2)	

**Table 2:** The expression correlation between Nox4 and IL-6 in NSCLCs

		Nox4 expression No(%)		
	Numbers	(−,+)	(++,+++)	*p* value
IL-6 expression No(%)	81	43(53.1)	38(46.9)	0.015
	71	25(35.2)	46(64.8)	

### IL-6 positively regulates NOX4 expression and activates PI3K/Akt pathway in A549 cells

**Figure 2:**
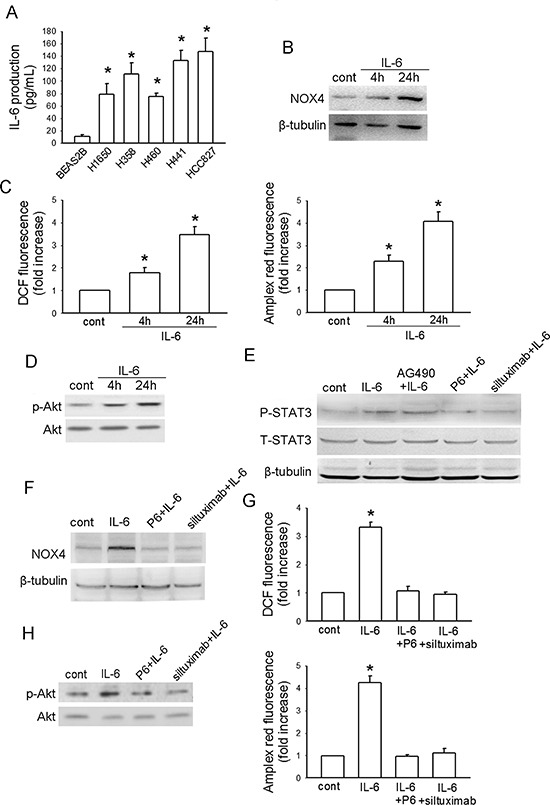
IL-6 stimulates NOX4/Akt pathway in A549 cells **(A)** ELISA analysis of IL-6 production in normal lung epithelial cells and cultured NSCLC cell lines. Bars are mean ± SD from four independent experiments. *Significantly different from BEAS2B cell group, *P* < 0.05. **(B-D)** The effects of IL-6 administration on NOX4 expression, ROS production and Akt activity in A549 cells at the indicated times, respectively. Bars are mean ± SD from four independent experiments. *Significantly different from control, *P* < 0.05. **(E)** The effect of IL-6 on STAT3 activity, and the influence of IL-6 neutralizing antibody siltuximab, JAKs inhibitor P6 (2.5 μM) or a selective JAK2 inhibitor of AG490 on IL-6-mediated STAT3 activation in A549 cells. **(F-H)** P6 or siltuximab could efficiently block the enhancement effects of IL-6 on NOX4 expression, ROS production and Akt activity in A549 cells after 48-hour incubation. Bars are mean ± SD from four independent experiments. *Significantly different from control, *P* < 0.05.

To dissect whether IL-6 stimulates NOX4/Akt signaling, we first examined the IL-6 production in NSCLC cell lines (A549, H460, H358, H441 and HCC827) and normal lung epithelial BEAS2B cells. The results showed that all the NSCLC cell lines and BEAS2B cells produced their own IL-6, and IL-6 production was markedly higher in NSCLC cell lines than that in the normal lung epithelial cells (Fig. 2A). Fig. 2B showed that IL-6 (10 ng/mL) treatment led to a time-dependent increase in NOX4 level in A549 cells. Besides, IL-6 could also enhance ROS production analyzed by DCF assay, as well as the preduction of superoxide and hydrogen peroxide analyzed by amplex red assay, respectively (Fig. 2C) and stimulate Akt activity (Fig. 2D) in a time-dependent manner in these cells.

Fig. 2E showed that IL-6 could stimulate STAT3 activity after 24-hour treatment, which was reversed by either IL-6 neutralizing antibody siltuximab (20 μg/mL) or JAKs inhibitor P6 (2.5 μM). However, consistent with another report [20], we found that AG490 (50 μM), a selective inhibitor of JAK2, had no influence on IL-6-induced STAT3 activation. Therefore, siltuximab and P6 were used for subsequent experiments. The results indicated that additional administration of siltuximab or P6 sufficiently blocked the enhancement effect of IL-6 on NOX4 expression (Fig. 2F) as well as ROS production (Fig. 2G) and Akt activity (Fig. 2H) after 48-hour incubation. Therefore, these data suggest that IL-6 can stimulate NOX4/Akt signaling via activation of JAK/STAT3 pathway.

### NOX4 enhances IL-6 production and activates IL-6/STAT3 signaling in A549 cells

**Figure 3:**
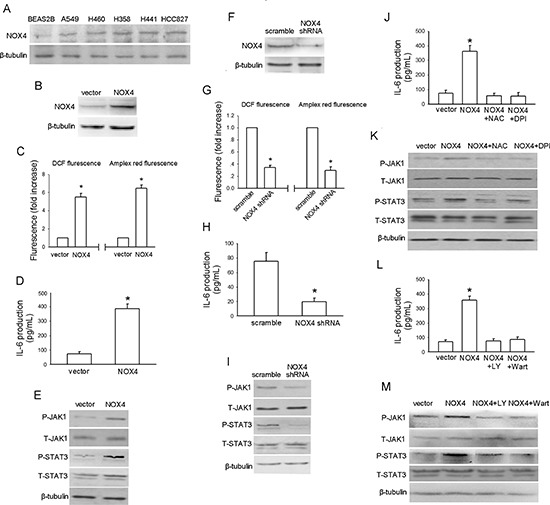
NOX4 stimulates IL-6 expression and JAK1/STAT3 activity in A549 cells via activation of PI3K/Akt pathway **(A)** Western blotting analysis of NOX4 expression in normal lung epithelial cells and cultured NSCLC cell lines. **(B)** A549 cells were stably transfected with control vector, NOX4 plasmid, respectively. Overexpression of NOX4 was confirmed by western blotting. **(C-E)** The effects of NOX4 overexpression on ROS production, IL-6 production and JAK1/STAT3 activity after 24 hours. Bars are mean ± SD from four independent experiments. *Significantly different from vector control, *P* < 0.05. **(F)** A549 cells were stably transfected with scramble shRNA, NOX4 shRNA, respectively. Knockdown of NOX4 was analyzed by western blotting. **(G-I)** Silencing NOX4 significantly inhibited ROS production, IL-6 production and JAK1/STAT3 activity after 24 hours. Bars are mean ± SD from five independent experiments. *Significantly different from vector control, *P* < 0.05. **(J-K)** Stably NOX4 overexpressing A549 cells were incubated with two common ROS scavengers including NAC (25 μM) and DPI (10 μM, an inhibitor of NADPH oxidase) for 24 hours, and then IL-6 levels and JAK1/STAT3 activity were determined. Bars are mean ± SD from four independent experiments. *Significantly different from vector control, *P* < 0.05. **(L-M)** Stably NOX4 overexpressing A549 cells were incubated with 30 μM of LY294002 or 10 μM of Wortmannin and control solvent for 24 hours, and then IL-6 levels and JAK1/STAT3 activity were determined. Bars are mean ± SD from four independent experiments. *Significantly different from vector control, *P* < 0.05.

To explore whether NOX4 enhances IL-6 expression in NSCLC cells as well, we first sought to determine the NOX4 expression phenotype in NSCLC cell lines (A549, H460, H358, H441 and HCC827) and normal lung epithelial BEAS2B cells. The results of western blotting assay revealed that NOX4 expression was markedly higher in NSCLC cell lines than that in the normal lung epithelial cells (Fig. 3A).

The impact of NOX4 on IL-6 expression in NSCLC cells was first evaluated in A549 cells stably expressing ectopic NOX4. The transfection efficiency was confirmed by western blotting (Fig. 3B). As shown in Fig. 3C, NOX4 overexpression substantially increased the total ROS levels, as well as the preduction of superoxide and hydrogen peroxide, respectively. Fig. 3D showed that overexpression of NOX4 significantly promoted IL-6 production in A549 cells assayed by ELISA. As a previous study indicated that JAK1 is the critical JAK kinase contributing to STAT3 activation and mediates IL-6-induced STAT3 activation in lung cancer cells [20], we next determined the effect of NOX4 overexpression on JAK1/STAT3 activity in A549 cells. As shown in Fig. 3E, NOX4-overexpressing A549 cells displayed much higher levels of phosphorylated JAK1 and STAT3 compared with vector control. To further confirm the role of NOX4 in regulation of IL-6 production and IL-6/STAT3 signaling in A549 cells, NOX4 expression was depleted with its specific shRNA (Fig. 3F). NOX4 knockdown could significantly reduced the ROS production in A549 cells determined by DCF and amplex red assay (Fig. 3G). As expected, NOX4 knockdown substantially suppressed IL-6 production (Fig. 3H) as well as JAK1/STAT3 signaling (Fig. 3I) in these cells.

ROS/PI3K/Akt pathway is the well-documented downstream signaling of NOX [21–22]. We used two common ROS scavengers including NAC (25 μM) and DPI (10 μM, an inhibitor of NADPH oxidase) with the doses selected according to our previous report [12]. Fig. 3J and 3K showed that NAC or DPI administration could efficiently block the enhancement effect of NOX4 on IL-6 expression, as well as JAK1/STAT3 activation in A549 cells, respectively. Treatment of A549 cells with highly selective PI3K/Akt pathway inhibitors, LY294002 (30 μM) or wartmannin (10 μM), could also block the effect of NOX4 on IL-6 production (Fig. 3L) and JAK1/STAT3 activity (Fig. 3M) in these cells. As MEK/Erk pathway is another important downstream signaling of NOX especially in vascular endothelial cells [23], we sought to explore whether MEK/Erk pathway is also involved in NOX4-stimulated IL-6/STAT3 signaling in A549 cells. The results showed that inhibition of MEK/Erk pathway by its specific inhibitor PD98509 (30 μM) had little or minimal effects on NOX4-mediated above effects in A549 cells (data not shown). Taken together, these findings suggest that NOX4 enhances IL-6 production and activates IL-6/STAT3 signaling in A549 cells mainly through stimulation of ROS/PI3K/Akt pathway.

### NOX4 and IL-6 promotes proliferation and survival of A549 cells

**Figure 4:**
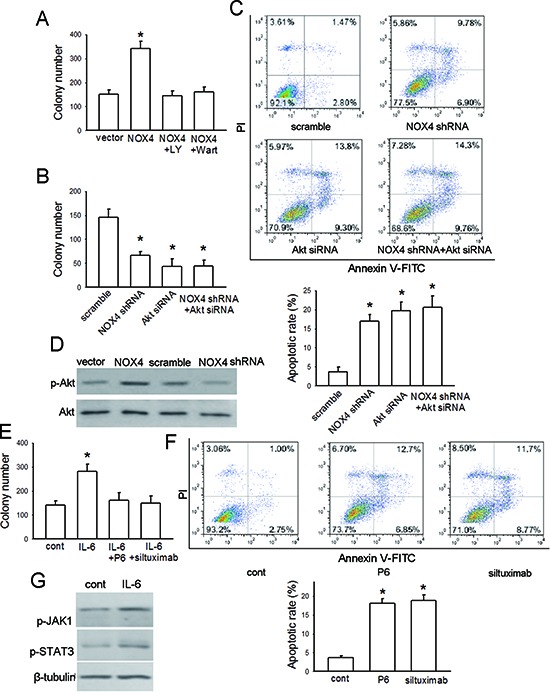
NOX4 and IL-6 promotes proliferation and survival of A549 cells **(A)** The effect of NOX4 overexpression on A549 cell proliferation, and effects of inhibition of PI3K/Akt pathway by LY294002 or Wortmannin on NOX4-promoted cell proliferation. The proliferation of cells was evaluated using colony formation assay. Bars are mean ± SD from three independent experiments. *Significantly different from vector control, *P* < 0.05. **(B-C)** A549 cells were transfected with NOX4 shRNA, Akt siRNA or NOX4 shRNA plus Akt siRNA, respectively. The proliferation of cells was evaluated using colony formation assay. Cell apoptosis was confirmed by flow cytometry analysis. Bars are mean ± SD from four to five independent experiments. *Significantly different from vector control, *P* < 0.05. **(D)** The effects of NOX4 overexpression or knockdown on Akt activity after 24 hours determined by western blotting. **(E)** The effect of IL-6 on cell proliferation, and the influence of IL-6 neutralizing antibody siltuximab or JAKs inhibitor P6 on IL-6-mediated proliferation in A549 cells. Bars are mean ± SD from four independent experiments. *Significantly different from control, *P* < 0.05. **(F)** The effects of siltuximab and P6 on cell apoptosis after 48-hour incubation. Bars are mean ± SD from five independent experiments. *Significantly different from control, *P* < 0.05. **(G)** The effects of IL-6 on JAK1/STAT3 activity determined by western blotting.

As Fig. 4A showed, NOX4 overexpression could substantially increase the anchorage-dependent growth of A549 cells. Treatment of NOX4-transduced A549 cells with a highly selective PI3K/Akt pathway inhibitor, LY294002 (30 μM), could block the enhancement effect of NOX4 on cell growth. Comparable data were also obtained from cells treated with another PI3K/Akt pathway inhibitor, wartmannin (10 μM). However, inhibition of MEK/Erk pathway by PD98509 (30 μM) had no significant effects on NOX4-mediated growth of A549 cells (data not shown). Further studies indicated that either depletion of NOX4 by its specific shRNA or knockdown of Akt by Akt siRNA substantially suppressed A549 cell growth, and when Akt expression was depleted, NOX4 shRNA had no additional inhibitory effect on A549 cell growth (Fig. 4B). Next we performed flow cytometry assay to determine the role of NOX4 in cell apoptosis. The results indicated that knockdown of either NOX4 or Akt increased A549 cell apoptosis after 48 hours, and NOX4 depletion had no additional enhancement effect on cell apoptosis compared with Akt knockdown alone (Fig. 4C). Fig. 4D showed that NOX4 overexpression significantly stimulated Akt activity, while NOX4 depletion caused a reduction in Akt activity in A549 cells. Therefore, these results demonstrate that NOX4 promotes A549 cell growth and survival via PI3K/Akt pathway-dependent manner.

Fig. 4E depicted that IL-6 (10 ng/mL) administration alone also substantially promoted the anchorage-dependent growth of A549 cells. Such effect was efficiently blocked by additional either P6 (2.5 μM) or siltuximab (20 μg/mL) treatment. The results obtained from flow cytometry assay indicated that either P6 (2.5 μM) or siltuximab (20 μg/mL) treatment could significantly increase A549 cell apoptosis after 48-hour incubation (Fig. 4F). Moreover, we found that IL-6 treatment resulted in a significant increase in both JAK1 and STAT3 activity in A549 cells after 24-hour incubation (Fig. 4G). These results together suggest that IL-6/STAT3 signaling can promote proliferation and survival of A549 cells.

### Functional interplay of NOX4 and IL-6/STAT3 pathway contributes to growth and survival of A549 cells

**Figure 5:**
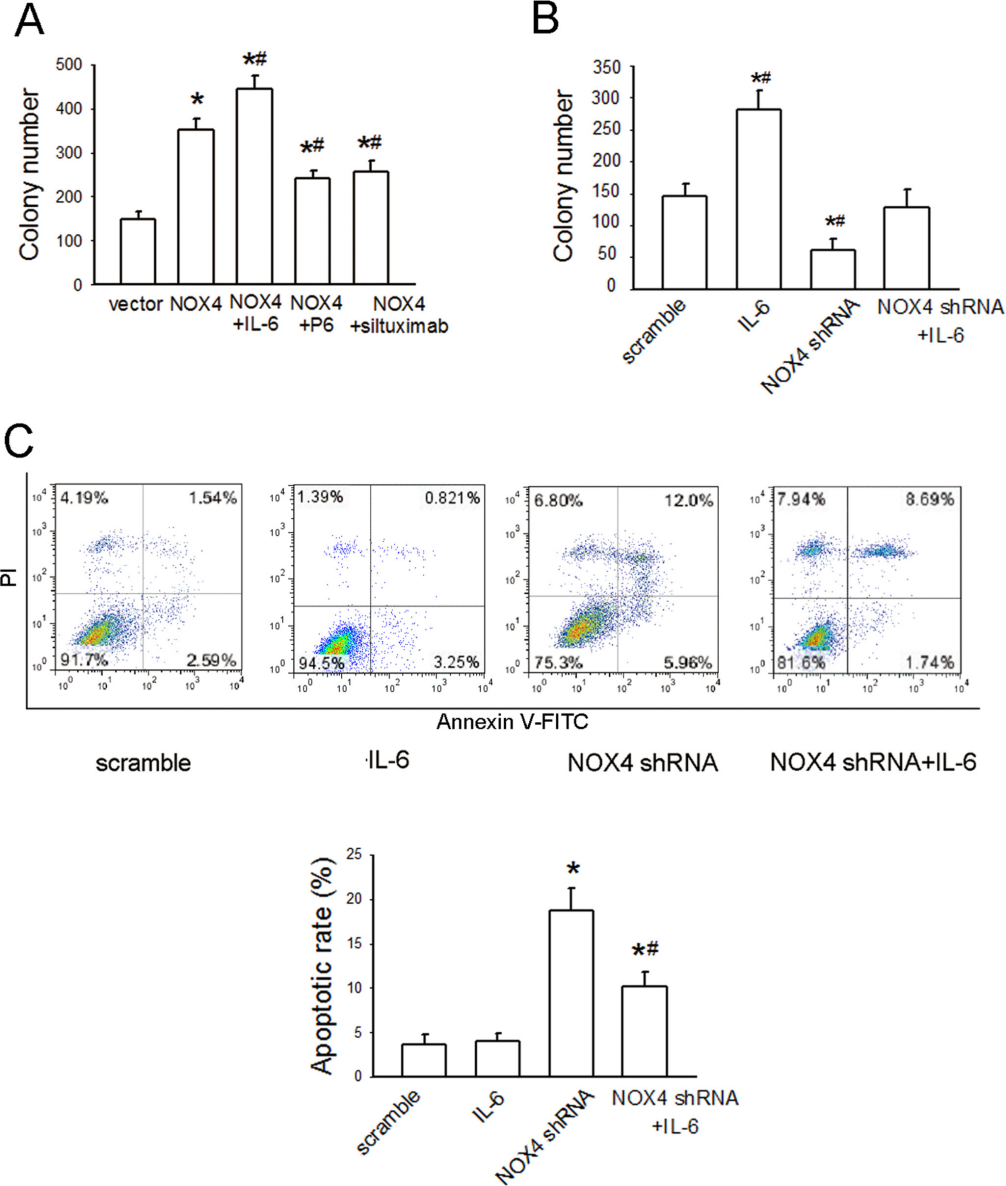
NOX4 interplays with IL-6 to regulate A549 cell proliferation and survival *in vitro* **(A)** The effects of IL-6, P6, or siltuximab on the proliferation of NOX4-transfected A549 cells. Bars are mean ± SD from four independent experiments. *Significantly different from control, *P* < 0.05. #Significantly different from NOX4 group, *P* < 0.05. **(B)** The effect of IL-6 on the proliferation of NOX4-depleted A549 cells. Bars are mean ± SD from four independent experiments. *Significantly different from control, *P* < 0.05. #Significantly different from NOX4 shRNA group, *P* < 0.05. **(C)** The effect of IL-6 on cell apoptosis of NOX4-depleted A549 cells. Bars are mean ± SD from four independent experiments. *Significantly different from control, *P* < 0.05. #Significantly different from NOX4 shRNA group, *P* < 0.05.

We further evaluated the biological significance of the reciprocal regulation between NOX4 and IL-6/STAT3 pathway in the growth and survival of A549 cells. As shown in Fig. 5A, NOX4 overexpression substantially promoted A549 cell growth. Additional IL-6 treatment in NOX4-transfected A549 cells led to a further increase in cell growth, whereas either P6 (2.5 μM) or siltuximab (20 μg/mL) could partially reverse NOX4-mediated growth-promoting effect in these cells. Furthermore, NOX4 depletion substantially suppressed cell growth, but additional IL-6 administration greatly rescued growth of NOX4-depleted cells (Fig. 5B). The data from flow cytometry assay also indicated that IL-6 administration could, at least partially, reverse the enhancement effect of NOX4 depletion on A549 cell apoptosis (Fig. 5C). Collectively, these results indicate that NOX4 and IL-6/STAT3 pathway are functionally interdependent in promoting A549 cell proliferation and survival.

### Reciprocal transcriptional activation between NOX4 and IL-6 contributes to growth and survival of another NSCLC cell line H460

**Figure 6:**
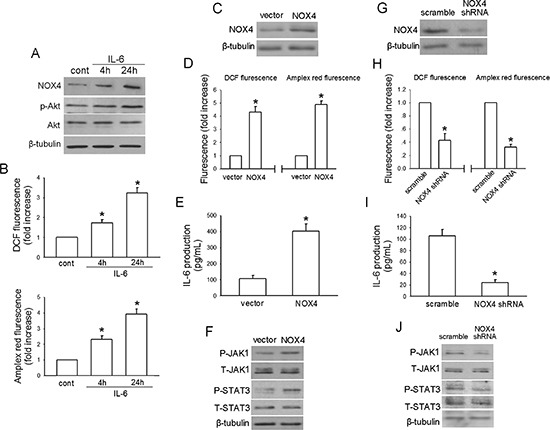
Reciprocal activation between IL-6/STAT3 and NOX4/Akt signalings in H460 cells **(A-B)** The effects of IL-6 administration on NOX4 expression, Akt activity and ROS production in H460 cells at the indicated times, respectively. Bars are mean ± SD from four independent experiments. *Significantly different from control, *P* < 0.05. **(C)** H460 cells were stably transfected with control vector, NOX4 plasmid, respectively. Overexpression of NOX4 was confirmed by western blotting. **(D-F)** The effects of NOX4 overexpression on ROS production, IL-6 production and JAK1/STAT3 activity after 24 hours. Bars are mean ± SD from four independent experiments. *Significantly different from vector control, *P* < 0.05. **(G)** H460 cells were stably transfected with scramble shRNA, NOX4 shRNA, respectively. Knockdown of NOX4 was analyzed by western blotting. **(H-J)** Silencing NOX4 significantly inhibited ROS production, IL-6 production and JAK1/STAT3 activity after 24 hours. Bars are mean ± SD from five independent experiments. *Significantly different from vector control, *P* < 0.05.

To exclude the possibility that the observed effects are restricted to A549 cells, we sought to confirm the above effects in another NSCLC cell line H460. Like in A549 cells, IL-6 (10 ng/mL) treatment led to time-dependent increase in NOX4 level (Fig. 6A), Akt activity (Fig. 6A) as well as ROS production (Fig. 6B) in H460 cells. We also found that NOX4 overexpression (Fig. 6C) could significantly enhance ROS production (Fig. 6D), IL-6 expression (Fig. 6E) as well as the activity of its downstream JAK1 and STAT3 (Fig. 6F), while depletion of NOX4 (Fig. 6G) resulted in reduced ROS production (Fig. 6H), IL-6 expression (Fig. 6I) as well as JAK1 and STAT3 activity (Fig. 6J) in H460 cells.

**Figure 7:**
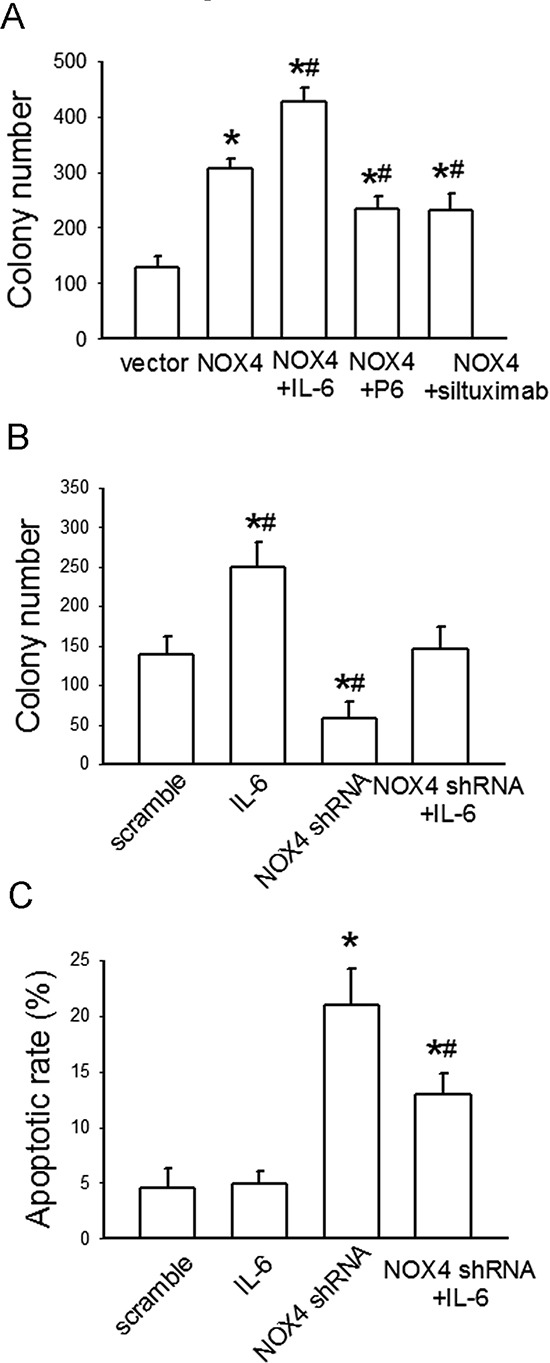
NOX4 interplays with IL-6 to regulate H460 cell proliferation and survival *in vitro* **(A)** The effects of IL-6, P6, or siltuximab on the proliferation of NOX4-transfected H460 cells. Bars are mean ± SD from four independent experiments. *Significantly different from control, *P* < 0.05. #Significantly different from NOX4 group, *P* < 0.05. **(B)** The effect of IL-6 on the proliferation of NOX4-depleted H460 cells. Bars are mean ± SD from four independent experiments. *Significantly different from control, *P* < 0.05. #Significantly different from NOX4 shRNA group, *P* < 0.05. **(C)** The effect of IL-6 on cell apoptosis of NOX4-depleted H460 cells. Bars are mean ± SD from four independent experiments. *Significantly different from control, *P* < 0.05. #Significantly different from NOX4 shRNA group, *P* < 0.05.

The functional interdependence between IL-6 and NOX4 in promoting H460 cell proliferation and survival was also examined. As shown in Fig. 7A, additional IL-6 treatment in NOX4-transfected H460 cells led to a further increase in cell growth compared with NOX4 transfection alone, whereas either P6 or siltuximab could partially reverse NOX4-mediated growth-promoting effect in these cells. Furthermore, additional IL-6 administration greatly rescued growth of NOX4-depleted cells (Fig. 7B). Flow cytometry assay results indicated that IL-6 administration could, at least partially, reverse the enhancement effect of NOX4 depletion on H460 cell apoptosis (Fig. 7C).

### Functional interplay of NOX4 and IL-6/STAT3 pathway in enhancing growth and survival of NSCLC cells *in vivo*

**Figure 8:**
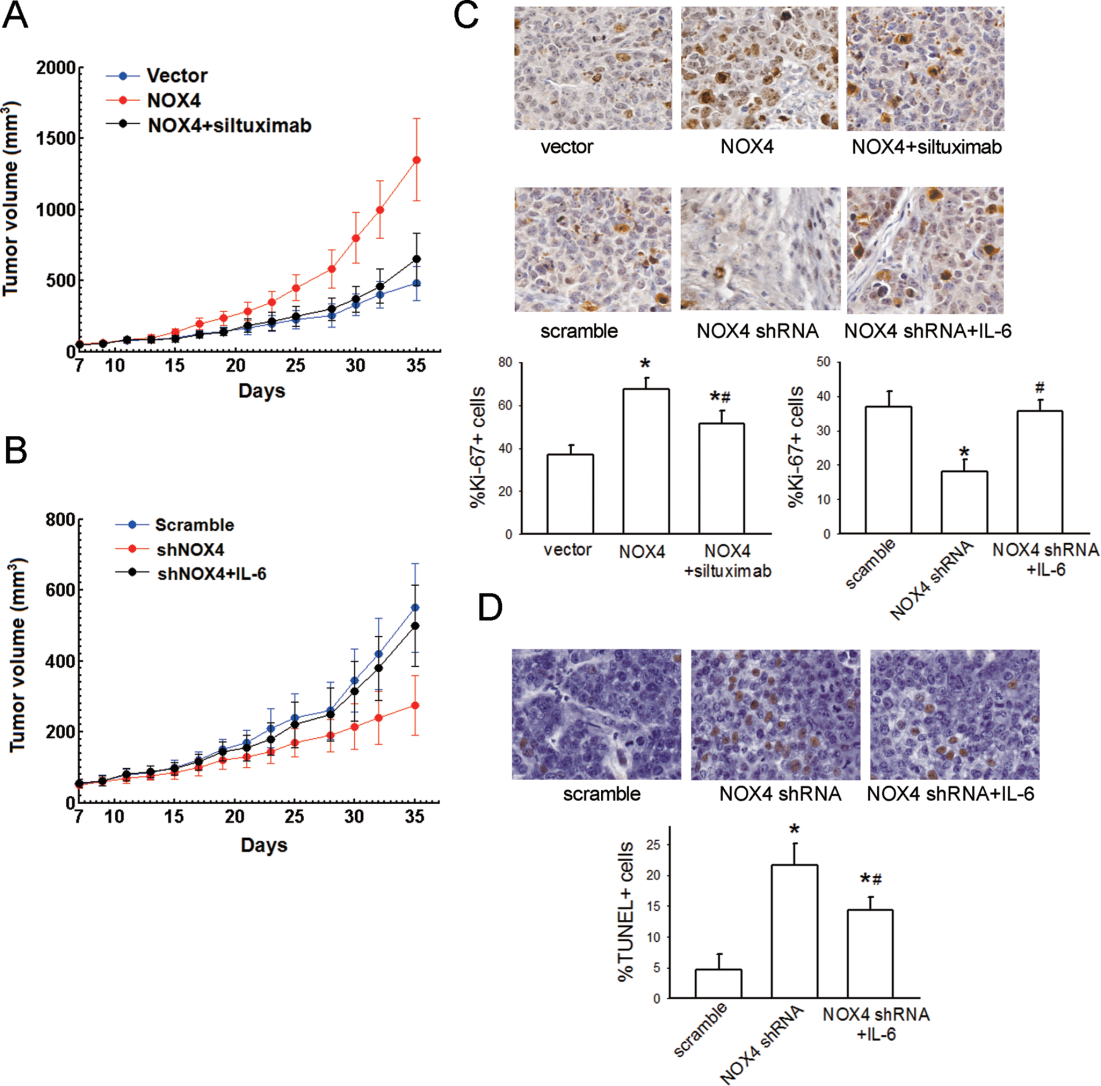
NOX4 interplays with IL-6 to regulate A549 cell proliferation and survival *in vivo* **(A-B)** A549 cells stably expressing NOX4 or its control vector, or stably transfecting NOX4 shRNA or its scramble shRNA were transplanted into nude mice (*n* = 10 per group), respectively. After seven days, siltuximab (10 mg/kg, twice a week, i. p.) or IL-6 (25 μg/Kg, three times a week, sc) was administrated, respectively. Tumor size was measured every 2 days for indicated period. The representative tumors and growth curves of tumor are shown. **(C)** The positive staining of Ki67 expression per field from paraffin-embedded sections of NOX-transfected A549 cells or those treated with siltuximab determined by immunohistochemistry and morphometric quantification (*n* = 10 per group). All scale bars represent 50 μm. *Significantly different from control, *P* < 0.05. #Significantly different from NOX4 or NOX4 shRNA group, respectively, *P* < 0.05. **(D)** Apoptotic nuclei with fragmented DNA were detected by TUNEL staining, and further analyzed by morphometric quantification. The final statistical graphs represent 10 non-overlapping images of each tumor specimens (*n* = 10 per group). *Significantly different from control, *P* < 0.05. # Significantly different from NOX4 shRNA group, *P* < 0.05.

We further confirmed the functional interplay of NOX4 and IL-6 in the tumorigenecity of NSCLC cells *in vivo*. A549 cells (approximately 1 × 10^6^ cells) were subcutaneously inoculated into the right flank of 6-week-old female nude mice. As shown in Fig. 8A, tumors formed by NOX4-transduced A549 cells grew faster than vector-control tumors, and treatment with siltuximab partially reversed such effect. Conversely, NOX4 shRNA-transfected A549 cells produced smaller tumors compared with scramble, and additional IL-6 administration could significantly rescue tumor growth (Fig. 8B). Furthermore, the functional interplay of NOX4 and IL-6 in enhancing growth and survival of NSCLC cells *in vivo* was also confirmed by Ki67 (the biomarker to evaluate tumor proliferation) (Fig. 8C) and TUNEL staining analysis (evaluation of tumor apoptosis) (Fig. 8D).

**Figure 9:**
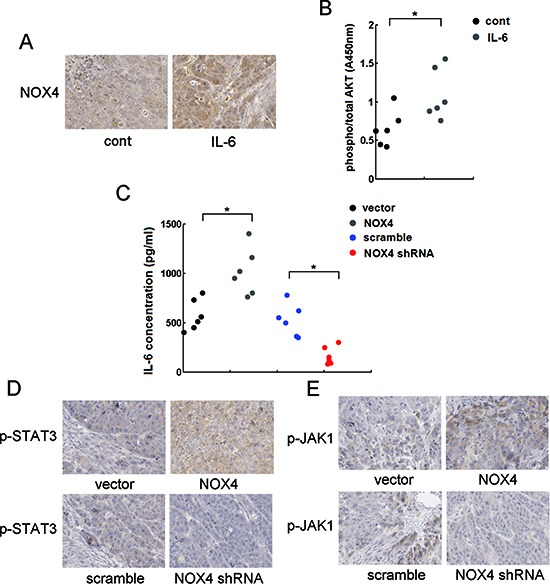
Reciprocal activation of NOX/Akt and IL-6/JAK1/STAT3 signalings *in vivo* **(A)** The effect of IL-6 on NOX4 expression *in vivo* analyzed by immunohistochemistry. **(B)** The effect of IL-6 on pAkt expression level, and the ratio of pAkt to total Akt was determined by ELISA assay. **(C)** The effects of NOX4 overexpression of NOX4 knockdown on IL-6 production in A549 tumor tissues assayed by ELISA. **(D-E)** The effects of NOX4 overexpression of NOX4 knockdown on p-STAT3 or p-JAK1 expression levels in A549 tumor tissues analyzed by immunohistochemistry. All scale bars represent 50 μm. *P < 0.05.

To explore the mechanism underlying the functional interplay of NOX4 and IL-6 in enhancing A549 tumor growth and survival of NSCLC cells *in vivo*, we sought to determine the reciprocal activation between NOX4/Akt and IL-6/STAT3 signalings *in vivo*. Fig. 9A showed that IL-6 treatment caused much more levels of NOX4 expression in vivo as determined by immunohistochemistry assay, as well as significantly enhanced levels of pAkt as determined by ELISA (Fig. 9B). On the other hand, the results obtained from ELISA assay showed that NOX4-transduced A549 cells produced more levels of IL-6 compared with control (Fig. 9C). Immunohistochemistry analysis showed that NOX4-transduced A549 tumors displayed higher activity of JAK1 and STAT3 (Fig. 9D and E). In contrast, NOX4 depletion resulted in lower levels of IL-6 and lower activity of JAK1 and STAT3 compared with control *in vivo* (Fig. 9C, D and E).

**Figure 10:**
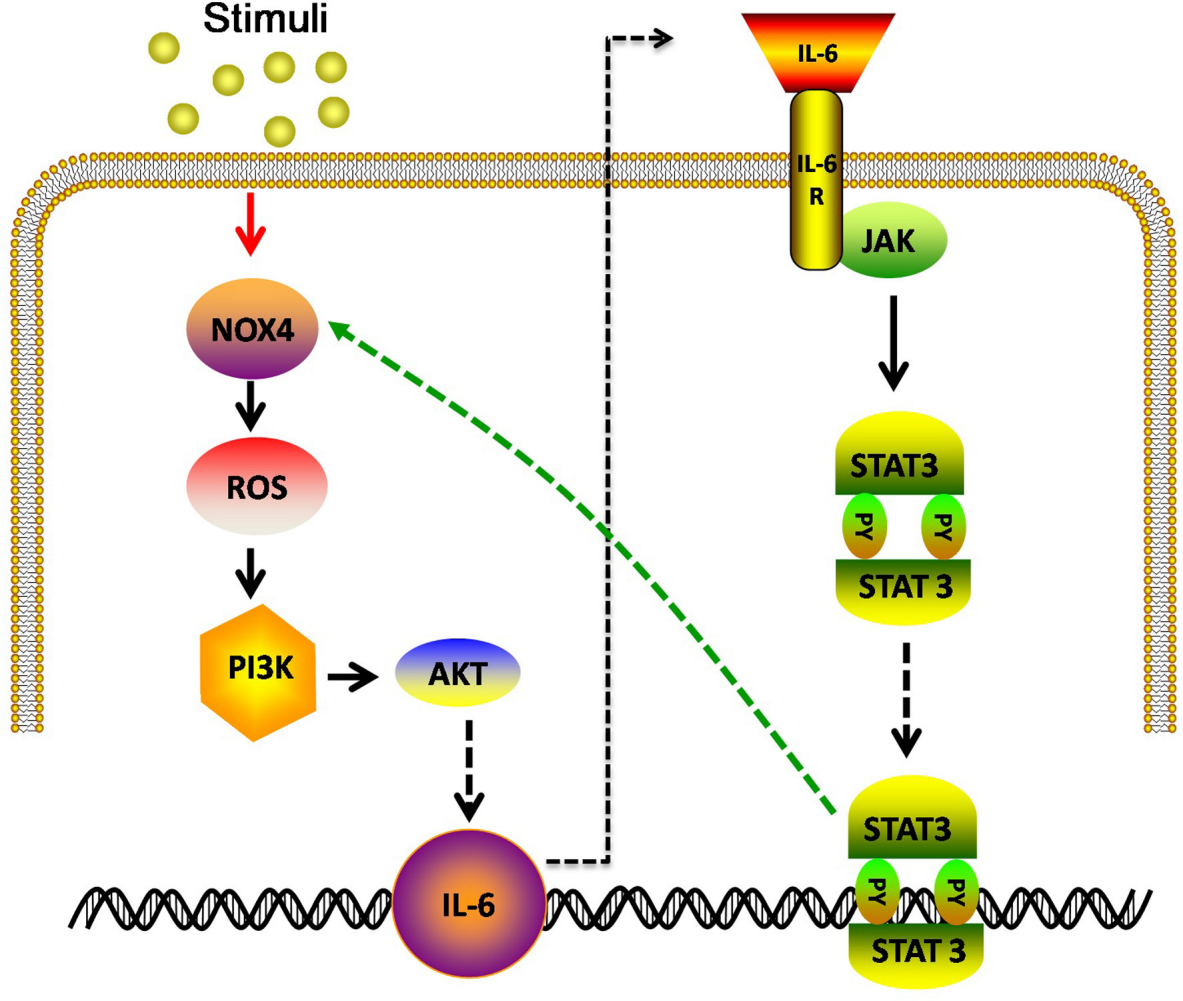
Model: IL-6 stimulates NOX4 expression and ROS/Akt signaling in NSCLC cells NOX4 also enhances IL-6 production and activates IL-6/STAT3 signaling in NSCLC cells.

Taken together, the above observations show that NOX4 and IL-6 reciprocally stimulate the expression of each other (Fig. 10), thus promoting proliferation and survival of NSCLC cells.

## DISCUSSION

Previous studies have shown that high level of IL-6 in circulation is a poor prognostic marker for survival of advanced NSCLC patients [15]. Furthermore, Haura et al. reported that both IL-6 and IL-6 receptor components gp80 and gp130 are abundantly expressed in NSCLC specimens [24]. In the present study, we found that the adjacent normal tissues of NSCLC had much lower levels of IL-6. Moreover, the clinical correlation studies showed that NOX4 levels are positively correlated with the IL-6 levels.

Tumor-promoting cytokines released by immune/inflammatory cells, including IL-6, act in a paracrine manner to activate transcription factors, such as NF-κB, STAT3 and AP-1, to achieve their effects in many types of cancer cells [25]. In this study, we found that exogenous IL-6 treatment could stimulate STAT3 activity in NSCLC cells via activation of JAK (but not JAK2). IL-6 treatment promoted NSCLC cell growth and survival mainly via JAK1/STAT3 pathway. Besides, IL-6 could also stimulate NOX4/ROS/Akt signaling via activation of JAK/STAT3 pathway, which is probably due to the direct activation of NOX4 promoter by STAT3 as observed in human aortic smooth muscle cells [26]. As the positive NOX4/Akt loop has been confirmed to promote NSCLC cell progression [19], the present study also indicates a novel mechanism underlying IL-6-mediated effects that involves activation of NOX4/Akt signaling in NSCLC cells.

Inflammatory cytokines in the tumor microenvironment can stimulate ROS accumulation in tumor cells, which implicates ROS in cytokines-mediated chronic inflammation-associated cancer [27]. However, the mechanisms remain largely unknown. This study combined with another report showing IFN-γ and LPS can synergistically induce Duox2/DuoxA2 expression and ROS production in human pancreatic cancer cells [6], strongly supports that NOXs are the major source of ROS production during inflammatory stimuli and act as a critical modulator of the inflammatory response in the context of tumorigenesis.

Similar with the study showing that NOX4-dependent ROS generation contributes to hypoxia-induced IL-6 production in renal cell carcinoma cells [18], in the present study, we found that NOX4 could enhance IL-6 expression and stimulate its downstream JAK/STAT3 signaling in NSCLC cells through stimulation of PI3K/Akt pathway. As IL-6/STAT3 signaling can promote proliferation and survival of NSCLC cells, these data suggest that NOX4-induced IL-6 expression, at least partly, accounts for its tumor-promoting effect. The exact mechanisms by which NOX4 stimulates IL-6 expression in NSCLC cells have not been explored. The possible explanation may be that NF-κB is a well-known downstream of Akt in cancer [28, 29], and NF-κB can directly bind to IL-6 promoter and subsequently activate IL-6 gene expression [30, 31].

Autocrine IL-6 have been confirmed in lung cancer cells and breast cancer cells [32]. Though activating mutations in epidermal growth factor receptor (EGFR) resultes in activate transcription of IL-6 [17], and activated Ras may also lead to IL-6 induction in lung cancer cells [33], the comprehensive mechanisms for IL-6 production in lung cancer cells remain unclear. The present study found that ectopic NOX4 expression significantly promoted IL-6 expression in NSCLC cells. This finding suggests that NOX4 upregulation during NSCLC development and progression, as another important factor, regulates IL-6 production in NSCLC cells, and undoubtedly, augments chronic inflammation-associated cancer.

IL-6 in the tumor microenvironment is one of the most critical tumor-promoting cytokines in various cancers including NSCLC [34, 35]. Besides, several types of cancer cells including breast cancer cells and lung cancer cells produce their own IL-6 [17, 36, 37], which acts in an autocrine manner to mediate cancer promotion. Following inflammatory stimulus, ROS accumulation in tumor cells promote cancer cell proliferation and survival through activation of various oncogenic signalings, including PI3K/Akt pathway and MAPK pathway [7, 38]. However, whether these factors have certain associations in tumor cells and cancer promotion are not well defined. In this study, we used recombined IL-6 to mimick microenvironmetal IL-6 and found that IL-6 treatment could significantly enhance NOX4 expression and ROS production in NSCLC cells. On the other hand, NOX4 overexpression could stimulate IL-6 expression in these cells. Therefore, these data indicate a novel NOX4-dependent link among IL-6 in the NSCLC microenvironment, oxidative stress in NSCLC cells and autocrined IL-6 in promoting NSCLC cell survival and proliferation.

Our study, both *in vitro* and *in vivo*, shows that NOX4 overexpression or IL-6 treatment alone results in enhanced growth and survival of NSCLC cells. Additional IL-6 treatment further enhances NOX4-promoted cell growth and survival, whereas blockade of IL-6 signaling renders impaired NOX4-mediated effects in these cells. Moreover, NOX4 depletion substantially suppressed cell growth, but additional IL-6 administration greatly rescued cell growth. These results indicate that either IL-6 or NOX4 contributes NSCLC cell promotion, and more importantly, the reciprocal positive regulation between NOX4 and IL-6 may mutually and interdependently reinforce NSCLC cell proliferation and survival. Therefore, combination therapy targeted both NOX4 and IL-6 may achieve potentially superior efficacy against NSCLC.

Our work has some limitations. It remains to be investigated to what extent the present principal finding can be generalized to NSCLC cell types other than those examined in this study and various species of human cancers. Besides, whether NOX4 is also involved in other inflammatory cytokines-mediated NSCLC promotion needs further exploration. Notwithstanding these limitations, this study does demonstrate that NOX4/Akt and IL-6/STAT3 signalings can reciprocally and positively regulate each other, leading to enhanced NSCLC cell proliferation and survival. Therefore, NOX4 may serve as a promising target against NSCLC alone with IL-6 signaling.

## MATERIAL AND METHODS

### Materials

Recombinant human IL-6 (Cat#8904) was purchased from Cell Signaling Technology Inc. The pyridine 6 (JAK inhibitor I or P6) and AG490 were obtained from EMD Chemicals. Siltuximab (CNTO328) was from Centocor Inc. Wartmannin and LY294002 (PI3K inhibitors) were obtained from Merck. Cell culture reagents were obtained from Invitrogen. All other reagents were from Sigma unless stated otherwise.

### Specimen preparation and immunohistochemical analysis

The clinical NSCLC tissues were obtained from Xiyuan hospital (Beijing, China). Prior patient consent and approval from the Ethics Committee of Xiyuan hospital were obtained for the use of clinical specimens and information for research purposes.

The surgical NSCLC specimens and matched adjacent normal tissues were fixed in buffered formalin (10% vol/formalin in water, PH 7.4) and embedded in paraffin wax. The specimens underwent immunohistochemical staining for analysis of NOX4 and IL-6 expression. The primary NOX4 and IL-6 antibodies (from Cell Signaling Technology Inc.) were applied to the slides and incubated at 4°C overnight. The slides were washed and then stained with the secondary antibody and DAB disclosure. The degree of immunostaining of paraffin-embedded sections was scored based on the intensity index of staining. The proportion of tumor cells was scored as follows:, 1 (< 10% postitve tumor cells), 2 (10%–50% positive tumor cells), and 3 (> 50% positive tumor cells). The intensity of staining was graded according to the following criteria: 0 (no staining); 1 (weak staining = light yellow), 2 (moderate staining = yellow brown), and 3 (strong staining = brown). The staining index was calculated as staining intensity score × proportion of positive tumor cells. A staining index of ≥ 6 (++, +++) was defined as high expression, while < 6 (–, +) was defined as low expression [39].

### Cell lines, plasmids, and transfection

Human NSCLC cell lines and normal lung epithelial cells (BEAS2B) were originally purchased from ATCC. Cells were cultured in Dulbecco's modified Eagle's medium (DMEM) supplemented with 10% fetal bovine serum (Gibco) and penicillin 100 (U/ml)/streptomycin (100 μg/ml). Stable cell lines expressing the NOX4 or shNOX4 were generated by transfection of pCMV-NOX4 or pRS-shNOX4 into A549 and H460 cells, according to our previous study [19]. For silencing Akt, Akt siRNA (Cat#sc-29195), and negative control siRNA were transiently transfected into A549 cells using Lipofectamine-2000 (Invitrogen) according to the manufacturer’ instruction. Cells were cultured for 48 h before harvest.

### Western blotting

Western blotting protocol was according to our previous report [40]. Briefly, cell lysates were separated by SDS/PAGE in 10% Tris-glycine gels (Invitrogen) and transferred to a nitrocellulose membrane. For analysis of NOX4, anti-NOX4 rabbit polyclonal antibody (ab154244; Abcam, Cambridge, MA, USA) diluted with 5% BSA to 1: 1000 was used. For analysis of Akt and p-Akt, blots were probed with their specific antibodies (diluted with 5% BSA to 1: 1000; all antibodies from Cell Signaling). Besides, antibodies against total STAT3, Y705-phosphoryl STAT3 (pY705-STAT3), total JAK1 and Y1022/1023-phosphoryl JAK1 (pY1022/1023-JAK1) were all obtained from Cell Signaling Technology and diluted with 5% BSA to 1: 1000 for experimental use. Membranes were probed with horseradish peroxidase (HRP)–labeled anti-rabbit secondary antibody (diluted with 5% BSA to 1: 1000, Cell Signaling). Antibody binding was detected by enhanced enhanced chemiluminescence detection kit (ECL) (UK Amersham International plc).

### ROS level assay

After IL-6 treatment, A549 or H460 cells (5×10^5^ cells/mL) were washed with cold PBS and incubated with DCFH-DA (Molecular Probes) at a final concentration of 5 μM or amplex red (Invitrogen) at a final concentration of 50 μM and 0.1 U horseradish peroxidase (HRP) (Sigma-Aldrich) in a total volume of 100 μl in 96-well microplates for 1 h at 37°C in darkness. The relative fluorescence intensity was determined using a fluorescence spectrophotometer (HITACHI, 650-60, Tokyo, Japan) with the excitation wavelength of 485 nm (DCFH-DA), 563 nm (amplex red) and the emission wavelength of 530 nm (DCFH-DA), 587 nm (amplex red).

### Colony formation assay

Cells were plated in 6-well plates (5×10^2^ cells per plate) and cultured for 14 days. The colonies were stained with 1% crystal violet for 10 mins after fixation with 10% formaldehyde for 5 mins. Colonies containing 50 or more cells were scored.

### Flow cytometry analysis of apoptosis

The protocol of flow cytometry analysis of apoptosis was according to our previous study [12]. After 48 hours of different treatments, A549 and H460 cells were collected, washed in cold PBS, and resuspended in 100 μl of 1× binding buffer with 3×10^5^ cells containing 5 μL of Annexin V-FITC (Pharmingen, San Diego, CA) and 5 μl of PI (Pharmingen, San Diego, CA).

### ELISA analysis

ELISA analysis for Akt, pAkt and IL-6 using the ELISA analysis kits (pAkt: Cat. no. 7160, CST; Akt: Cat. no. 7170, CST; IL-6: Cat. no. D6050, R&D systems) according to the manufacturer's instructions. For analysis Akt, pAkt and IL-6 *in vivo*, tumors were snapped frozen and homogenized in cell lysis buffer (CST; Cat. no. 9803).

### Xenograft studies

Animal handling procedures were approved by the Institutional Animal Care and Use Committee of Guangdong pharmaceutical university. A549 cells (approximately 1 × 10^6^ cells) were subcutaneously inoculated into the right flank of 6-week-old female nude mice. Seven days later, the nude mice were treated with siltuximab (10 mg/kg, twice a week, i. p.) or IL-6 (25 μg/Kg, three times a week, sc). Tumor sizes were calculated with the formula: (mm^3^) = (L × W^2^) × 0.5. The tumor volume was measured every other day.

### Ki67 staining and Tunel staining

The protocols of Ki67 staining and Tunel staining of tumor sections in mice were strictly according to our previous study [41].

### Statistical analysis

Statistical analysis was evaluated by Student's test for simple comparisons between two groups and one-way ANOVA for comparisons among multiple groups using JMP7.0 software (SAS Institute Inc, Cary, US). Pearson chi-square test was applied in studying the correlation between NOX4 expression and IL-6 level as well as clinicopathologic characteristics of NSCLC. All data are expressed as mean ± S.D. *P* value of < 0.05 was considered statistically significant.
